# Salusins: Potential Use as a Biomarker for Atherosclerotic Cardiovascular Diseases

**DOI:** 10.1155/2013/965140

**Published:** 2013-10-22

**Authors:** Kengo Sato, Rena Watanabe, Fumiko Itoh, Masayoshi Shichiri, Takuya Watanabe

**Affiliations:** ^1^Laboratory of Cardiovascular Medicine, Tokyo University of Pharmacy and Life Sciences, 1432-1 Horinouchi, Hachioji, Tokyo 192-0392, Japan; ^2^Department of Endocrinology, Diabetes and Metabolism, Kitasato University School of Medicine, 1-15-1 Kitasato, Minami-Ku, Sagamihara, Kanagawa 252-0374, Japan

## Abstract

Human salusin-**α** and salusin-**β** are related peptides produced from prosalusin. Bolus injection of salusin-**β** into rats induces more profound hypotension and bradycardia than salusin-**α**. Central administration of salusin-**β** increases blood pressure via release of norepinephrine and arginine-vasopressin. Circulating levels of salusin-**α** and salusin-**β** are lower in patients with essential hypertension. Salusin-**β** exerts more potent mitogenic effects on human vascular smooth muscle cells (VSMCs) and fibroblasts than salusin-**α**. Salusin-**β** accelerates inflammatory responses in human endothelial cells and monocyte-endothelial adhesion. Human macrophage foam cell formation is stimulated by salusin-**β** but suppressed by salusin-**α**. Chronic salusin-**β** infusion into apolipoprotein E-deficient mice enhances atherosclerotic lesions; salusin-**α** infusion reduces lesions. Salusin-**β** is expressed in proliferative neointimal lesions of porcine coronary arteries after stenting. Salusin-**α** and salusin-**β** immunoreactivity have been detected in human coronary atherosclerotic plaques, with dominance of salusin-**β** in macrophage foam cells, VSMCs, and fibroblasts. Circulating salusin-**β** levels increase and salusin-**α** levels decrease in patients with coronary artery disease. These findings suggest that salusin-**β** and salusin-**α** may contribute to proatherogenesis and antiatherogenesis, respectively. Increased salusin-**β** and/or decreased salusin-**α** levels in circulating blood and vascular tissue are closely linked with atherosclerosis. Salusin-**α** and salusin-**β** could be candidate biomarkers and therapeutic targets for atherosclerotic cardiovascular diseases.

## 1. Introduction

Biomarkers mostly consist of circulating proteins, peptides, or enzymes whose levels provide independent diagnostic and/or prognostic value by reflecting an underlying disease state. Studies to unravel novel biomarkers have attracted attention, especially in the field of cardiovascular medicine which warrants earlier diagnosis, risk stratification, and monitoring of therapeutic efficacy [1]. A number of candidate biomarkers have been extensively evaluated, but only a limited number have demonstrated significant diagnostic and/or therapeutic impact. High-sensitivity C-reactive protein, for example, is associated in part with risk of future cardiovascular morbidity and mortality among patients at high risk or with documented coronary artery disease (CAD) [2]. Deeper insights into the pathophysiology of atherosclerosis have successfully unraveled additional novel biomarkers for CAD [3, 4]. Proatherogenic vasoactive agents, such as angiotensin II, urotensin II, serotonin, and oxidized low-density lipoprotein (oxLDL), have been nominated as potential biomarkers for CAD [5–7]. Moreover, reduced circulating levels of antiatherogenic vasoactive agents, such as adiponectin and heregulin-*β*_1_, could also be used as indicators and/or negative risk factors for CAD [8, 9].

 Hypertension is a well-known risk factor for CAD. Hypertension-induced mechanical stimuli, such as pressure overload, stretch, and shear stress, cause arterial endothelial injury, leading to the development of atherosclerosis [10]. Potent vasoconstrictors, such as angiotensin II, urotensin II, and serotonin, all of which are agonists for G-protein-coupled receptors, play key roles as mediators linking hypertension and atherosclerosis [10]. These agents stimulate vascular inflammation and oxidative stress-induced endothelial injury, monocyte-endothelial adhesion, macrophage foam cell formation, and vascular smooth muscle cell (VSMC) migration and proliferation, which are the pivotal phenomena of atherosclerosis [11–15]. Of all historical candidate biomarkers extensively studied so far, the recently identified bioactivities of salusins on atherogenesis are of special note. Salusins, initially identified with their rapid hypotensive and bradycardic effects, have been recognized to exert multifold comprehensive influences on the development of atherosclerosis [16].

 This review focuses on the recent developments in salusin research, novel regulators of systemic hemodynamics and atherosclerosis, and discusses their emerging roles as promising biomarkers and therapeutic targets for atherosclerotic cardiovascular diseases.

## 2. Biosynthesis of Salusins

Bioinformatics analyses of a full-length enriched cDNA library originally derived from human cells allowed the prediction that sequences corresponding to an alternative splicing product of the torsion dystonia-related gene (TOR2A) could give rise to a precursor of two potential endogenous bioactive peptides, salusins [17]. This precursor, termed preprosalusin, has 242-amino acid residues and generates the 216-amino acid prosalusin after the removal of the N-terminal 26 amino acid signal peptide [17]. Proteolytic processing of prosalusin at the prosalusin C-terminal results in biosynthesis of two related peptides of 28 and 20 amino acids in length designated as salusin-*α* and salusin-*β* [17]. Salusin-*β* contains more hydrophobic amino acids residues than salusin-*α*, and they each have distinct physicochemical properties [17]. Although each salusin likely binds to its respective cell surface binding sites, their specific receptors have not been identified [18].

 Salusins are expressed and synthesized ubiquitously within human, rat, and mouse tissues, including the vasculature, central nervous system, and kidneys [17, 19, 20]. Expression of salusins is increased in renal tissues in fructose-load rats, an insulin resistance model [21]. Salusins are present in human plasma and urine [22, 23], indicating their possible role as peptide hormones in humans. Preprosalusin is expressed at high levels in human VSMCs and endothelial cells (ECs) [17]. Human monoblastic leukemia cell lines, such as THP-1 and U937, secrete salusin-*β* [24]. Secretion of salusin-*β* is stimulated by inflammatory cytokines, such as tumor necrosis factor-*α* and lipopolysaccharide; this response was markedly enhanced after these cells were induced to differentiate into macrophages by stimulation with phorbol 12-myristate 13-acetate [24]. An *in vivo* study demonstrated that salusin-*β* is produced in fibroblasts of the media in the aorta and VSMCs of the media in the left internal mammary artery and saphena in CAD patients during coronary artery bypass graft operations [25].

 In murines, however, prosalusin has a shorter amino acid length, and its processing is speculated to result in biosynthesis of the 18 amino acid putative salusin-*α*. The sequence has little homology with the 28 amino acid residue human salusin-*α*. In rat tissues, salusin-*α*-like immunoreactivity was detected by an antibody that specifically recognized rat salusin-*α* sequence but was not detected by an anti-human salusin-*α* antibody [19]. This could account for the minimal hemodynamic effects induced by human salusin-*α* peptide when intravenously injected into rats [17].

## 3. Systemic Hemodynamic Activities of Salusins

Initial studies indicated salusins as multifunctional regulators of hemodynamics [17]. Salusin-*β*-like immunoreactivity is localized in vasopressin-expressing neurons of the rat posterior pituitary and hypothalamus, which tempted us to speculate its neural secretion into the systemic circulation via axon terminals [26]. Indeed, salusin-*β* stimulates the release of arginine-vasopressin and oxytocin from the rat neurohypophysis [17, 27]. However, the magnitude of its release from the neuroendocrine system and subsequent contribution to systemic hemodynamics remain unknown. Bolus injection of salusin-*β* induces rapid and temporary hypotension and bradycardia by a cholinergic mechanism in addition to its direct negative inotropic effects [17, 28] but has no vasodilatory effect on isolated intact or deendothelialized vascular strips [17]. On the other hand, microinjection of salusin-*β* into the paraventricular nucleus increases blood pressure via the release of norepinephrine and arginine-vasopressin in renovascular hypertensive rats [29]. Salusins promote growth and induce hypertrophy of rat cardiomyocytes [30] and also exert antiapoptotic effects in these cells [31]. The negative inotropic effect on ventricular myocytes by salusin-*β* is ascribed by its inhibitory effect on action potentials and ionic currents in rats [32, 33].

## 4. Detection of Salusins in Biological Fluids

**Figure 1:**
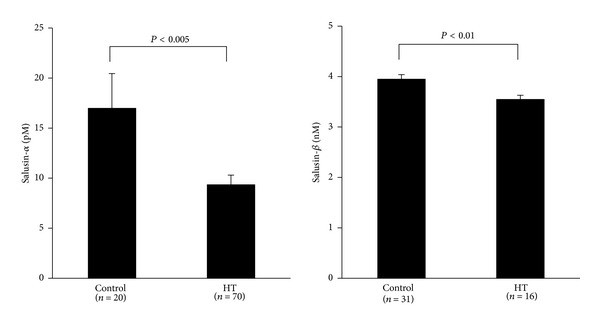
Comparison of circulating levels of salusin-*α* and salusin-*β* between normotensive subjects and hypertensive (HT) patients.

Initially, salusin-*α* has been successfully measured by radioimmunoassay in serum and urine [23]. However, the establishment of a salusin-*β* assay system faced extreme difficulty because of its unique physicochemical features [34]. Salusin-*β* easily and tightly adheres to polypropylene, polystyrene, and glass, which are major components of plastic tubes, tips, and containers essential for laboratory handling of peptides. Thus, salusin-*β* concentrations may be rapidly reduced to very low levels immediately after high-purity peptides are reconstituted and diluted in such commonly used experimental tubes [34]. We have recently resolved this problem by using a very small amount of organic solvent [22] and succeeded in measuring salusin-*β* concentrations in a variety of human biological fluids by enzyme-linked immunosorbent assay [35]. Our preliminary data show that circulating levels of salusin-*β* are higher than those of salusin-*α* and show significantly but slightly lower levels in patients with essential hypertension than healthy volunteers (Figure 1). Salusin-*α*, on the other hand, shows distinctly lower levels in essential hypertension.

## 5. Effects of Salusins on Inflammatory and Adhesion Molecules in ECs

**Figure 2:**
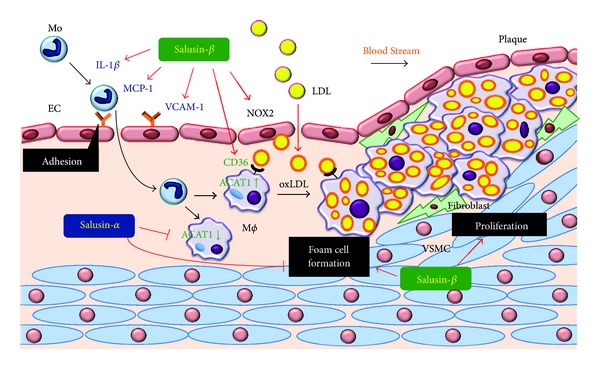
Modulatory effects of salusin-*α* and salusin-*β* on atherogenesis in vascular cells. Mo = monocyte, M*ϕ* = macrophage.

A recent study demonstrated a stimulatory effect of salusin-*β* on proinflammatory and oxidative stress molecules together with its intracellular mechanisms of action. Salusin-*β* induces the expression of interleukin-1*β* (IL-1*β*), monocyte chemoattractant protein-1 (MCP-1), vascular cell adhesion molecule-1 (VCAM-1), and NADPH oxidase 2 (Nox2), a potent source of reactive oxygen species, in human umbilical vein ECs (HUVECs) [36] (Figure 2). Further, salusin-*β* stimulates the adhesion of THP-1 monocytes onto HUVECs via nuclear factor-*κ*B (NF-*κ*B)-mediated VCAM-1 induction [36] (Figure 2). However, salusin-*α* does not exert similar effects (Table 1). Infusion of antisalusin-*β* antiserum into LDL receptor-deficient mice attenuated the induction of VCAM-1, MCP-1, and IL-1*β* as well as nuclear translocation of NF-*κ*B in aortic ECs, resulting in the prevention of monocyte adhesion to aortic ECs *in vivo* [36]. These data explain some of the mechanisms underlying the potent proatherosclerotic effects induced by salusin-*β*.

## 6. Effects of Salusins on Macrophage Foam Cell Formation

The overwhelming proatherosclerotic effect by salusin-*β* is its ability to induce macrophage foam cell formation. Macrophage foam cell formation is characterized by modified LDL, such as oxLDL and acetylated LDL, and subsequent cholesterol ester accumulation in macrophages. The intracellular free cholesterol level is increased by the endocytic uptake of oxLDL and acetylated LDL via CD36 and scavenger receptor class A (SR-A), respectively, and is decreased by efflux of free cholesterol mediated by ATP-binding cassette transporter A1 (ABCA1) [9]. Since excessive accumulation of free cholesterol is toxic to cells, free cholesterol must be either removed through efflux to extracellular acceptors, such as apolipoprotein (apo) A-I and high-density lipoprotein, or esterified to cholesterol ester by the microsomal enzyme acyl-CoA:cholesterol acyltransferase 1 (ACAT1) [9]. Our studies revealed that salusin-*α* and salusin-*β* induce the opposite effects on foam cell formation [37] (Table 1). Salusin-*α* suppresses while salusin-*β* stimulates foam cell formation and ACAT1 expression in primary cultured human monocyte-derived macrophages (Figure 2), and the regulation of ACAT1 expression by salusins is mediated via the G-protein/c-Src/PKC/MAPK pathway [37]. However, neither SR-A nor ABCA1 expression is affected by salusin-*α* and salusin-*β* in human monocyte-derived macrophages [37] (Table 1).

## 7. Effects of Salusins on VSMC and Fibroblast Proliferation

Similar to many other mitogenic peptides such as endothelin-1 and adrenomedullin [38–40], salusin-*β* stimulates proliferation of rat and human VSMCs and fibroblasts with the activation of immediate early response genes, *c*-*myc* and *fos* [17] (Figure 2). Salusin-*β* antagonizes the apoptotic death of cardiomyocytes [31], again sharing similar characteristics to other mitogenic peptides. Salusin-*β* stimulates the generation of intracellular cAMP in rat and human VSMCs. In contrast to salusin-*β*, salusin-*α* has marginal mitogenic effects in these cells [17] (Table 1). Therefore, salusin-*β* locally produced in the cardiovascular system could act on adjacent cells to increase VSMC and fibroblast cell numbers and cardiomyocyte hypertrophy.

## 8. Effects of Salusins on Atherosclerotic Lesion Development 

Contrasting modulatory effects of salusin-*α* and salusin-*β* on atherosclerotic lesion formation have also been demonstrated in *in vivo* atherosclerosis models: apoE-deficient mice and LDL receptor-deficient mice. Expression of salusin-*β* is increased in atherosclerotic lesions in LDL receptor-deficient mice [41]. Subcutaneous injection of salusin-*β* into LDL receptor-deficient mice aggravated atherosclerotic lesions, and this effect was associated with significantly increased serum LDL cholesterol levels [41]. Chronic infusion of salusin-*β* into apoE-deficient mice significantly enhanced atherosclerotic lesions in the aorta and macrophage infiltration into the lesions without affecting blood pressure or serum total cholesterol and glucose levels [42]. Further, neutralization of endogenous salusin-*β*  by chronic infusion of antisalusin-*β* antiserum into apoE-deficient mice prevented the development of atherosclerotic lesions. On the contrary, chronic infusion of salusin-*α* significantly suppressed aortic atherosclerotic lesions accompanied by a significant decrease in macrophage infiltration [42]. These data support the notion that salusin-*β* exerts systemic proatherogenic activity while salusin-*α* has a contrasting antiatherogenic effect.

 Foam cell formation induced by oxLDL is significantly increased in exudate peritoneal macrophages obtained from apoE-deficient mice infused with salusin-*β* but is decreased in those infused with salusin-*α* [42]. In macrophages of animals chronically infused with either salusin-*α* or salusin-*β*, ACAT1 expression is reduced or increased, respectively [42]. Macrophages of salusin-*β*-treated mice cause an increase in CD36 and SR-A but not ABCA1. These results obtained from *in vivo* treatment models are apparently inconsistent with those reported with primary cultured human monocyte-macrophages in which treatment with salusin-*α* or salusin-*β* for seven days did not affect SR-A expression [42]. This discrepancy may be attributed to the duration for which monocytes/macrophages are exposed to salusins. Taken together, the data indicate important roles for salusins as endogenous modulators of atherogenesis.

## 9. Neointimal Expression of Salusin-***β*** after Coronary Angioplasty

Coronary stent implantation in the porcine heart induces the formation of thick neointima histologically indistinguishable from human restenotic neointima. To investigate the expression of salusin-*β* in the restenotic neointima, bare metal stents were implanted into the left anterior descending coronary arteries of porcine hearts for 28 days, and the stented arteries were immunohistochemically examined. Salusin-*β* was expressed at high levels in small neovessels, especially in the macrophages and proliferative VSMCs around stent struts in the neointima [16]. In addition, a recent study showed a suppressive effect of salusin-*β* on angiogenesis that serves as collateral circulation to rescue ischemic myocardium after ischemia-reperfusion injury by transient coronary occlusion in rats [43]. These findings indicate that salusin-*β* promotes to aggravate myocardial ischemia following coronary events. 

## 10. Presence of Salusins in Atherosclerotic Lesions in Human Coronary Arteries

Immunohistochemical analyses of human coronary arteries from patients with acute coronary syndrome have shown the presence of immunoreactive salusin-*α* and salusin-*β* in fatty streaks and atheromatous plaques [37]. Salusin-*β* was expressed at high levels in macrophage foam cells, VSMCs, and fibroblasts within atherosclerotic lesions in coronary arteries, while the expression of salusin-*α* was at trace levels [37]. The preferential expression of endogenous salusin-*β* supports its role as an accelerator in the formation of human atherosclerotic lesions.

## 11. Linkage of Salusins with Atherosclerotic Cardiovascular Diseases

**Figure 3:**
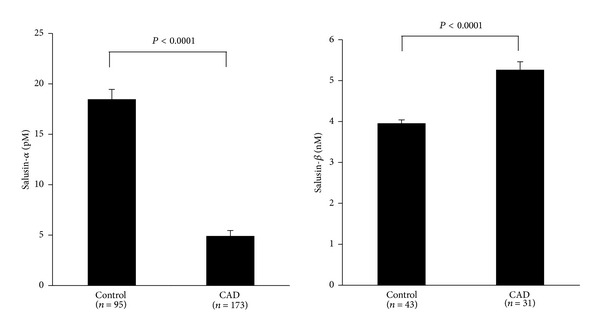
Comparison of circulating levels of salusin-*α* and salusin-*β* between non-CAD subjects and CAD patients.

As described above, our group has recently succeeded in determining human plasma levels of salusin-*β* [35]. Our data demonstrated that plasma levels of salusin-*β* were significantly higher in 31 patients with angiographically proven CAD than those levels in 43 subjects without CAD (Figure 3). On the other hand, serum salusin-*α* levels were significantly lower in 173 patients with angiographically proven CAD than in 95 non-CAD subjects [37] (Figure 3). Of these, patients with CAD showed the lowest levels of serum salusin-*α*, which were reduced in accordance with the severity of atherosclerotic lesions on coronary arteriography, and they were significantly lower in patients with triple-vessel disease than in patients with single-vessel disease [37]. Data from another group supports the view that serum salusin-*α* levels are inversely correlated with the presence and severity of CAD [44]. 

**Figure 4:**
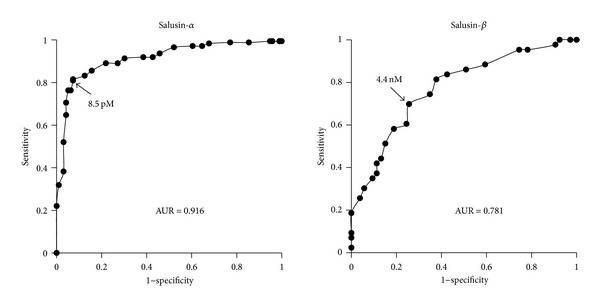
ROC curves of salusin-*α* and salusin-*β* for detecting CAD.

Receiver operating characteristic (ROC) curves were plotted, and the area under the curve (AUC) was analyzed to compare the predictive power of salusin-*α* and salusin-*β*. The optimal cut-off values of salusin-*α* and salusin-*β* for detecting CAD were set at the point showing a higher true-positive rate (sensitivity) with a low false-positive rate (1-specificity) on the respective ROC curve. The AUC values of salusin-*α* and salusin-*β* were 0.916 and 0.781, respectively (Figure 4). The cut-off levels were 8.5 pM for salusin-*α* and 4.4 nM for salusin-*β*, with sensitivities of 82% and 70% and specificities of 93% and 75%, respectively. Therefore, reduced levels of serum salusin-*α* could be a reliable biomarker for detecting CAD rather than increased salusin-*β*.

 Our data and others have demonstrated that decreased levels of serum salusin-*α* are associated with carotid atherosclerosis and cardiac dysfunction in patients with essential hypertension and with renal dysfunction in patients with chronic kidney diseases [45–47]. We recently suggested the possible mechanisms responsible for decreased production of salusin-*α* in atherosclerotic lesions. Salusin-*α* expression may be inhibited by the activation of Jak-2 kinase, which is expected to be upregulated by angiotensin II, inflammatory cytokines, and growth factors in atherosclerotic and hypertensive diseases [48].

## 12. Conclusions

These findings suggest that salusin-*α* and salusin-*β* show contrasting effects on atherosclerosis; salusin-*α* and salusin-*β* possess antiatherogenesis and proatherogenesis, respectively. Therefore, salusin-based treatments could emerge as a new line of therapy against atherosclerosis and its related diseases. Moreover, salusins may be clinically useful for the early detection of atherosclerotic cardiovascular diseases. Decreased salusin-*α* and/or increased salusin-*β* in the circulating blood and cardiovascular tissues could be a promising candidate biomarker for predicting atherosclerotic cardiovascular diseases.

## Figures and Tables

**Table 1:** Atherogenic effects of salusins on human vascular cells.

	Salusin-*α*	Salusin-*β*	Reference
EC			
IL-1*β* expression	→	↑	[36]
MCP-1 expression	→	↑	[36]
ICAM-1 expression	→	↑	[36]
VCAM-1 expression	→	↑	[36]
Monocyte			
Adhesion to EC	→	↑	[36]
Macrophage			
Foam cell formation	↓	↑	[37]
ACAT1 expression	↓	↑	[37]
SR-A expression	→	→	[37]
ABCA1 expression	→	→	[37]
VSMC			
Proliferation	*↗*	↑	[17]
Fibroblast			
Proliferation	*↗*	↑	[17]

Arrows indicate the stimulatory (↑, *↗*), suppressive (↓), or negative (→) effects listed in the right column.
